# Uncontrolled Confounders May Lead to False or Overvalued Radiomics Signature: A Proof of Concept Using Survival Analysis in a Multicenter Cohort of Kidney Cancer

**DOI:** 10.3389/fonc.2021.638185

**Published:** 2021-05-27

**Authors:** Lin Lu, Firas S. Ahmed, Oguz Akin, Lyndon Luk, Xiaotao Guo, Hao Yang, Jin Yoon, A. Aari Hakimi, Lawrence H. Schwartz, Binsheng Zhao

**Affiliations:** ^1^ Department of Radiology, Columbia University Irving Medical Center, New York, NY, United States; ^2^ Department of Radiology, Memorial Sloan Kettering Cancer Center, New York, NY, United States; ^3^ Department of Surgery, Memorial Sloan Kettering Cancer Center, New York, NY, United States

**Keywords:** radiomics, quality control, machine learning, TCGA, The Cancer Imaging Archive (TCIA), clear cell renal cell cancer

## Abstract

**Purpose:**

We aimed to explore potential confounders of prognostic radiomics signature predicting survival outcomes in clear cell renal cell carcinoma (ccRCC) patients and demonstrate how to control for them.

**Materials and Methods:**

Preoperative contrast enhanced abdominal CT scan of ccRCC patients along with pathological grade/stage, gene mutation status, and survival outcomes were retrieved from The Cancer Imaging Archive (TCIA)/The Cancer Genome Atlas—Kidney Renal Clear Cell Carcinoma (TCGA-KIRC) database, a publicly available dataset. A semi-automatic segmentation method was applied to segment ccRCC tumors, and 1,160 radiomics features were extracted from each segmented tumor on the CT images. Non-parametric principal component decomposition (PCD) and unsupervised hierarchical clustering were applied to build the radiomics signature models. The factors confounding the radiomics signature were investigated and controlled sequentially. Kaplan–Meier curves and Cox regression analyses were performed to test the association between radiomics signatures and survival outcomes.

**Results:**

183 patients of TCGA-KIRC cohort with available imaging, pathological, and clinical outcomes were included in this study. All 1,160 radiomics features were included in the first radiomics signature. Three additional radiomics signatures were then modelled in successive steps removing redundant radiomics features first, removing radiomics features biased by CT slice thickness second, and removing radiomics features dependent on tumor size third. The final radiomics signature model was the most parsimonious, unbiased by CT slice thickness, and independent of tumor size. This final radiomics signature stratified the cohort into radiomics phenotypes that are different by cancer-specific and recurrence-free survival; HR (95% CI) = 3.0 (1.5–5.7), p <0.05 and HR (95% CI) = 6.6 (3.1–14.1), p <0.05, respectively.

**Conclusion:**

Radiomics signature can be confounded by multiple factors, including feature redundancy, image acquisition parameters like slice thickness, and tumor size. Attention to and proper control for these potential confounders are necessary for a reliable and clinically valuable radiomics signature.

## Introduction

Tumor radiomics is a rapidly evolving field aiming to link tumor imaging phenotypes to pathological and clinical outcomes in a quantitative and non-invasive way (1). Radiomics generally converts medical image data into a large-scale and mineable set of imaging features, termed radiomics features, that characterize tumor imaging phenotypes (2). Radiomics signatures, essentially constellations of radiomics features, have shown to be helpful in plenty of medical tasks (3), including predicting malignancy in lung nodules at lung cancer screening CT scans (4), predicting genomic alteration on lung cancer imaging (5), predicting tumor recurrence and patients’ survival (6), and assessing response to treatment (7, 8).

Radiomics signature models have been developed by cancer researchers but their usefulness is usually difficult to replicate at other institutions or cohorts. This is mostly due to challenges encountered in the construction of a radiomics signature models attributed to radiomics feature redundancy and image quality differences (resulting from differences in image acquisition/technical parameters or from scanner vender differences). Another challenge facing useful radiomics signature is the need to provide new information independent of already known and established prognosticators, especially tumor size which is retrieved from routine clinical imaging without the need to run radiomics image analysis (9). Feature redundancy is a challenge to replicate and consolidate radiomics signatures. Two research teams, Lu et al. (10) and Berenguer et al. (11), independently pointed out that radiomics feature sets, which usually contain several hundreds to a thousand radiomics features, could actually be summarized into dozens of representative features. The variations in image acquisition parameters, *e.g.* thin/thick slice thickness and sharp/smooth reconstruction kernels, etc., could produce images of different qualities (12), which might impede generalization of radiomics signatures. For instance, the performance of radiomics signature developed using CT images of thin slice thickness decreased when applied on CT images with thicker in the predicting the risk of malignancy of lung nodule (13) and cancer-related genomic mutation status (14). Finally, including tumor size measurement (unidimensional, bidimensional and three dimensional) within radiomics features creates confusion about the usefulness of the texture based radiomics; it raises the question whether the prognostic or predictive radiomics signature effect is mainly driven by tumor size which is readily available through routine medical imaging without the need for radiomic analysis. Association between radiomics signature and well-established clinical factors (*e.g.*, tumor size or patient’s age), may lead to overvalued radiomics signatures; this is because the predictive value of radiomics signature may be exaggerated by radiomics’ association with these important clinical factors (15).

Several approaches were proposed for establishing reproduceable and generalizable radiomics studies including radiomics reporting guidelines, such as Radiomics Quality Score (RQS) (9), The Image Biomarker Standardization Initiative (IBSI) (16, 17), and recently harmonization algorithms (18), such as Combat. Although these studies have demonstrated that radiomics signature could be impacted by multiple clinical and technical factors, there is still suboptimal awareness of this confounding potential and lack of consensus on how to control for such confounding. For example, within the RQS, although imaging protocol was suggested to be reported, it does not provide a reliable statistical method to control the confounding effect from imaging protocol and does not alarm that confounding effect of imaging protocol could lead to fake result. In IBSI, its main focus is on standardizing implementation parameters for radiomics feature extraction instead of controlling confounding effect. For those harmonization algorithms, like Combat, although they showed promising potential on removing confounding effect, however, there is limitation on application on new data. For example, when new data were added, the new data have to be combined with original data and the harmonization has to be re-established on the entire combined database (19).

Therefore, in this study, we designed multiple radiomics signature models to show the effect of uncontrolled confounders which may lead to false/overvalued radiomics signature among patients with clear cell renal cell carcinoma (ccRCC). The reason for using radiomics analysis on ccRCC as an example is that, ccRCC is the predominant pathological subtype (85%) in renal adenocarcinomas which account for 90% of kidney cancers because of its variable course (20, 21). The prediction of survival outcomes for ccRCC patients still remain challenging (22–25), due to the variation in ccRCC’s growth pattern, with some tumor showing an indolent growth pattern while others exhibiting aggressive behaviors including local recurrence after resection and distant metastases (26, 27).

## Method

We aimed to conduct this study in The Cancer Genome Atlas—Kidney Renal Clear Cell Carcinoma (TCGA-KIRC) cohort data (28) which is a publicly available dataset from multiple medical institutions in the US. The TCGA-KIRC project house the pathological, clinical, and imaging data for patients with clear cell renal cell carcinoma (ccRCC).

Compared to single-center data, the TCGA-KIRC was a multicenter data therefore was more heterogenous in terms of tumor’s pathological stage and grade as well as the image acquisition parameters. The Cancer Imaging Archive (TCIA) (29) represent a repository of clinical imaging for patients/tumors included in the TCGA cohort housing de-identified clinical imaging and provide a great resource for researchers to conduct and validate their imaging related studies.

**Figure 1:**
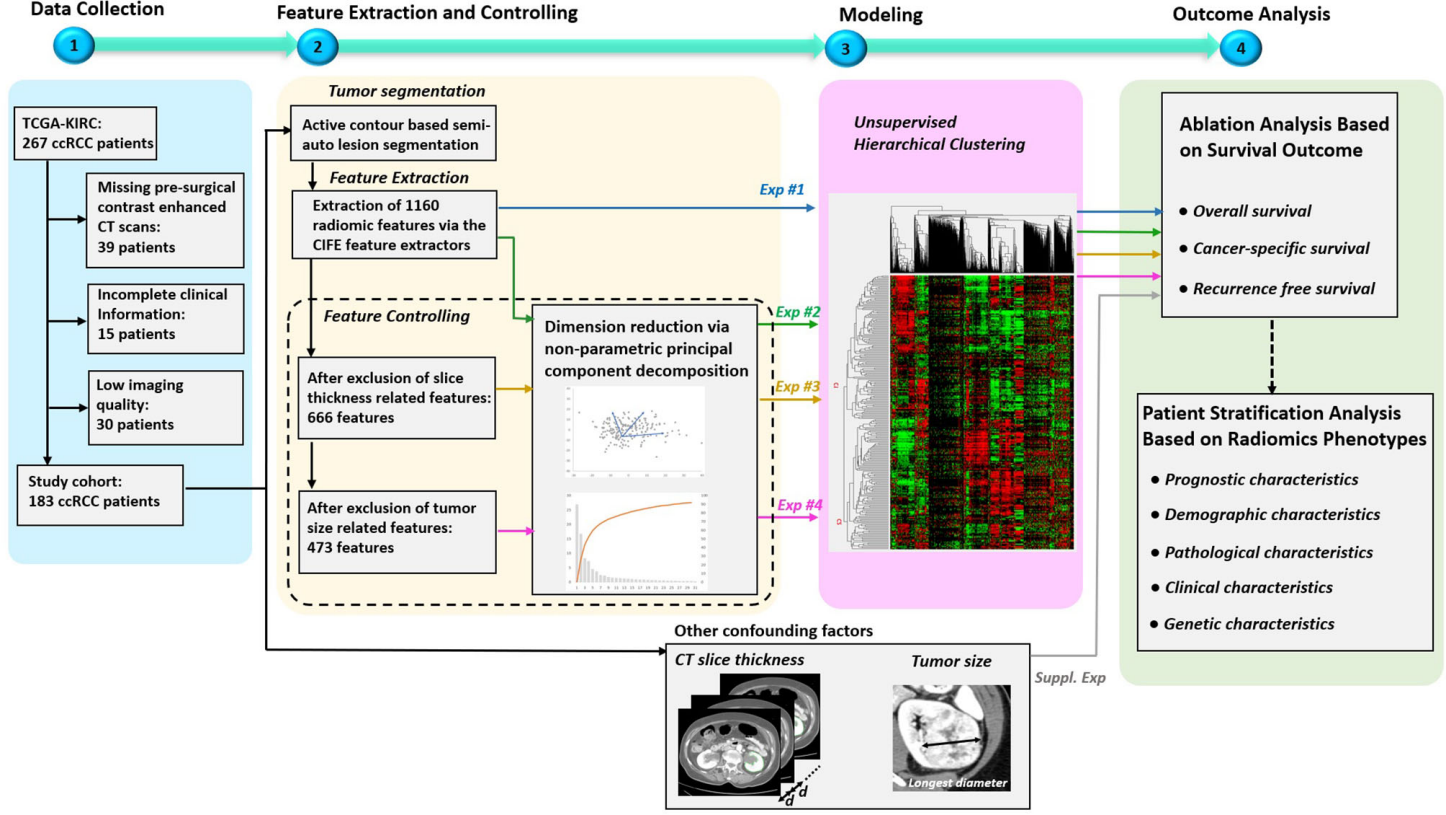
Overview of the study design. The study mainly consisted of four key parts: 1) patient data collection, 2) feature extraction and controlling, 3) Modeling, and 4) Outcome analysis. Specially, four experiments were designed to evaluate the effects on radiomics signatures built by radiomics feature sets under four different controlling levels. In addition, supplementary experiments were performed to explore the association between outcomes and the confounding factors, such as CT slice thickness and tumor size.

The overview of our study design is presented in **Figure 1**. Our study design followed the basic radiomics phases, which included data collection, feature extraction, modeling, and outcome analysis (9, 17). The highlights of the study are the following. First, all the used data are publicly available in the TCIA, so that other researchers can easily and reliably replicate our results. Second, multiple factors that might affect radiomics analysis (9) were investigated, including feature redundancy (*e.g.* correlation among features), image acquisition parameters (slice thickness was the main CT parameter impacting radiomic signature), and signature’s dependency to tumor size (a previously validated prognostic factor). Four radiomics signatures were successively built throughout our study that included: 1) entire radiomics feature set, 2) radiomics feature set after dimension reduction (i.e. excluding redundant features), 3) radiomics feature set after further exclusion of radiomics features affected by CT scan slice thickness, and 4) radiomics feature set after further exclusion of tumor size related features. Third, to address the over-fitting problem, non-parametric principal component decomposition (PCD) for dimension reduction and unsupervised hierarchical clustering for pattern discovery were used. Fourth, the radiomics signatures associations with clinical outcomes (OS, PFS, and RFS) were tested using Kaplan–Meier’s analysis. Finally, supplementary analyses were conducted to illustrate how impacting factors can affect the radiomics signatures.

### Patient Data Collection

The data we used were downloaded from the TCGA-KIRC project, which is publicly available in the TCIA dataset (https://wiki.cancerimagingarchive.net/display/Public/TCGA-KIRC, accessed in August 2016). It contained 267 ccRCC patients collected from multiple medical centers nationwide. The downloaded content contained both presurgical contrast-enhanced abdominal CT scans and clinical information such as pathological stage, grade, gene mutation status, and patient’s survival outcomes. For the survival outcome, patients were censored at their last follow-up date if: they were alive (overall survival-OS), alive or dead from non-ccRCC related illness (cancer-specific survival-CSS), alive without tumor recurrence (recurrence-free survival-RFS). Somatic gene mutation status of our patients was retrieved from TCGA official website and adjudicated at Memorial Sloan Kettering Cancer Center (MSKCC) based on collaborative clinical TCGA (cTCGA) consortium data (30). Genetic information included gene mutation status of VHL, PBRM1, SETD2, BAP1, and KDM5C genes. All patients’ informed consents and institutional review boards’ approvals were obtained as part of the TCGA/TCIA efforts, and all demographic and imaging data were de-identified to comply with the Health Insurance Portability and Accountability Act (HIPAA).

In our study, 183 out of 267 patients were included in the final segmentation and analysis (as shown in **Figure 1**) based solely on the availability of pre-surgical contrast-enhanced abdominal CT scans where ccRCC was depicted and segmented to generate radiomics features.

### Tumor Segmentation and Feature Extraction

A fellowship trained abdominal radiologist performed ccRCC segmentations. The radiologist was blinded to the various study endpoints (pathology at surgery and patient outcomes). For image analysis we used a MatLab (MathWorks, Natick, Massachusetts) based dedicated software application to visualize and segment the tumor from each patient’s CT scan. This semi-automated algorithm, combining the region-based active contours and a level set approach, was used in a slice-by-slice fashion (i.e. the entire ccRCC tumor was segmented) (31). The initial step for tumor segmentation required the radiologist to manually select a region-of-interest (ROI) that roughly enclosed the tumor region on a single CT slice. Rough boundary localization of the tumor was then automatically generated by the software algorithm and propagated to consecutive slices, serving as an initial ROI for subsequent segmentations on the neighboring images. The final tumor segmentation boundaries were then verified and fine-tuned by the radiologist (32). The total volume of the tumor (created by adding all segmentations from all slices) was then utilized to generate the radiomics features of each individual tumor.

A total of 1,160 radiomics features, i.e., quantitative imaging features, were extracted from each segmented tumor *via* the Columbia Image Feature Extractor (CIFE) (33) which has been successfully applied in many radiomics studies (34–36). More details of the CIFE, as well as its comparison with two other open-source feature extractors, the IBEX (37) and Pyradiomics (38), can be founder at (33). Three preprocesses were performed before the feature extraction, 1) a modified soft tissue CT window was adopted with level of 50 HU and width of 175 HU, 2) voxel resolution was resampled to 0.5 × 0.5 × 0.5 mm³ and 3) image was discretized into 64 bins.

### Principal Component Decomposition (PCD)

In this study, we introduced an unsupervised method, PCD (39), for feature dimension reduction. On contrary to its supervised counterpart, an unsupervised method focuses more on the intrinsic characteristics of features and is not easily affected by the overfitting problem. PCD belongs to a type of non-parametric transformation that is able to convert a set of possibly correlated features into a set of linearly uncorrelated variables. Such uncorrelated variables are called principal components and are ranked by their corresponding variance, which is their contribution to feature variability in the data. Hence, in the set of resulting principal components, the first principal component has the largest variance, and each succeeding component in turn has a smaller variance. We hypothesized that if a principal component had a larger variance, it would contain more information, so that the dimension reduction could be fulfilled by selecting a compact set of principal components that had the large variance while excluding a large number of principal components with small variances (i.e. excluding those with the least input to the data). In this study, Matlab version 9.5 was used. Principal components that summed up to 99% contribution to the total variance were selected as the new representative features.

### Exclusion of Slice Thickness Related Features

The main CT scan parameter in this cohort that affected (was associated with) radiomics features values was the CT scan slice thickness (among other parameters including CT scan voltage (kVp), vender and reconstruction algorithm). Because this is a multi-institutional cohort with different imaging protocols, we aimed to remove the potentially confounding effect of slice thickness from the radiomics signature to be built. The identification of radiomics features dependent on slice thickness involved three steps: First, the patients were distributed into two groups: one with thin CT slices (i.e. ≤3 mm) and one with thick slices (i.e. >3 mm) (The selection of 3 mm as an cutoff is based on clinical practice (40)). Second, C-index (41) was calculated for each feature based on the slice thickness group labels. The C-index, in this model, provided a measure of how good a radiomics feature could fit a binary outcome (groups of slice thickness ≤3 mm and >3 mm). In other words, we attempted to measure how much of the radiomics feature was explained by the CT slice thickness. Generally, for C-index, values below 0.5 indicate poor fitting, values over 0.7 indicate good fitting, values over 0.8 indicate strong fitting, and a value of 1 means perfect fitting. In radiomics signature model #3, we excluded all radiomics features whose C-index was >0.8 in order to remove the radiomics features that are heavily influenced/biased by CT slice thickness.

### Exclusion of Tumor Size Related Features

In this project, we aimed to build a radiomics signature that deliver new prognostic information, independent of tumor size which has long been known as an important prognosticator. The correlation between tumor size and the radiomics features were measured by Pearson’s linear correlation coefficients (also called Pearson’s R). In our study, tumor size was obtained by measuring the longest diameter across the tumor’s cross-sectional region, as shown in **Figure 1**. The features that have strong positive or negative correlation with tumor size (Pearson’s R >0.7 or <−0.7, p < 0.05) were excluded from radiomics signature model #4 as tumor size dependent features.

### Unsupervised Clustering

Unsupervised hierarchical clustering was used to identify the clusters of ccRCC tumors based on input radiomics features. We identified two major clusters of ccRCC tumors in this study. During the clustering, the distance between two clusters in the feature space was measured by ‘cosine’ distance. The unsupervised clustering method was intrinsically an iteration process based on the similarity among radiomics features. At each iteration of the clustering, two of the most similar clusters were combined into one cluster and then acted as one cluster for the next iteration. A cluster node in the clustering tree could be one individual radiomics features or several radiomics features. A detailed description of unsupervised hierarchical clustering can be found in our previous publication (10).

### Association Between Confounding Factors and Survival Outcomes

In this study, a direct association between confounding factors and survival outcomes was also studied. The two confounding factors were CT slice thickness and tumor size. The information of CT slice thickness was retrieved from DICOM attributes tagged as (0018,0050). Patients were assigned to two subgroups with slice thickness ≤3 mm (74 patients) and >3 mm (109 patients). With respect to tumor size, patients were assigned to two subgroups with tumor size less than or equal to the median size value (60 patients) and greater than the median value (123 patients).

### Statistical Analysis

Unsupervised clustering and principle component analyses were used to stratify the cohort into two groups/phenotypes. The association of this radiomics clustering/phenotypic binary classification was tested primarily with survival outcomes (OS, CSS and RFS) using Kaplan–Meier curves and Cox-regression models. Secondarily, the radiomics cluster’s association with other patient’s and tumor’s characteristics (including demographic characteristics (age, gender and race), pathological characteristics (tumor grade), American Joint Committee on Cancer tumor, node, metastasis staging (AJCC TNM staging), and genetic characteristics (VHL, PBRM1, SETD2, BAP1, and KDM5C) using Chi-Square and T-test when appropriate. P-values smaller than 0.05 indicated statistical significance. All statistical analyses were performed using Matlab 2020a.

## Results

### Patient Characteristics and CT Examination

A total of 183 patients were included in our study according to the inclusion and the exclusion criteria. The patient characteristics are presented in **Table 1**. Patients’ average age was 60 years (± standard deviation (std) of 12). Majority of patients were men (66%) and white (96%). The mean ± std of tumor size was 6.4 ± 3.2 cm. The minimum and maximum of tumor size were 1.5 and 15.5 cm, respectively. The cohort was close to be evenly split between early stage (52% had stage I) and advanced stage (48% has stages II–IV). The CT scan characteristics are presented in **Table 2**. Most of the patients were scanned by the same vender CT scanner (GE Medical System, 85%) but with different slice thicknesses; 60% had thin CT slices scans while 40% had thick CT slices scans. A more detailed CT characteristics were provided in **Supplement S2**.

**Table 1:** Patient characteristics.

Patient characteristics	Total patients (n = 183)
**Age, year**	**59.9 ( ± 11.7)**
**Gender**	** **
** Female**	**62 (34%)**
** Male**	**121 (66%)**
**Race**	** **
** White**	**176 (96%)**
** Others**	**7 (4%)**
**Tumor grade**	** **
** G1**	**1 (1%)**
** G2**	**72 (39%)**
** G3**	**79 (43%)**
** G4**	**31 (17%)**
**AJCC TNM staging**	** **
** Stage I**	**96 (52%)**
** Stage II**	**14 (8%)**
** Stage III**	**48 (26%)**
** Stage IV**	**25 (14%)**
**Distant Metastasis**	** **
** M0**	**160 (87%)**
** M1**	**23 (13%)**
**VHL mutation**	** **
** Positive**	**100 (55%)**
** Negative**	**71 (38%)**
** Not available**	**12 (7%)**
**PBRM1 mutation**	
** Positive**	**52 (28%)**
** Negative**	**119 (65%)**
** Not available**	**12 (7%)**
**SETD2 mutation**	
** Positive**	**14 (8%)**
** Negative**	**157 (86%)**
** Not available**	**12 (7%)**
**BAP1 mutation**	
** Positive**	**16 (9%)**
** Negative**	**155 (85%)**
** Not available**	**12 (7%)**
**KDM5C mutation**	
** Positive**	**8 (4%)**
** Negative**	**163 (89%)**
** Not available**	**12 (7%)**

Values are presented as n (%) for categorical variables and mean ( ± std) for continuous variables.

**Table 2:** CT scan characteristics.

CT scan characteristics		Total patients (n = 183)
**Scanner manufacturer**		** **
** **	**GE Medical System**	**156 (85%)**
** **	**SIEMENS**	**24 (13%)**
** **	**Philips**	**3 (2%)**
**CT slice thickness**		** **
** **	**thin section (≤3 mm)**	**109 (60%)**
** **	**thick section (>3 mm)**	**74 (40%)**
** **	**overall**	**3.63 ± 1.51, 1.25, 7.5**
**Current-time product (mAs)**		**324 ± 124, 101, 686**
**Pixel spacing (mm)**		**0.81 ± 0.10, 0.59, 0.97**
**Voltage (kVp)**		** **
** **	**120**	**172 (94%)**
** **	**130 or 140**	**11 (6%)**

Values are presented as frequency (%) for categorical variables and mean ± std, minimum and maximum for continuous variables.

### Ablation Analysis Based on Survival Outcome

As shown in **Figure 1**, radiomics-based analysis consisted of four experiments. The results of the four corresponding experiments are presented in **Table 3**.

**Table 3:** Results of the four designed experiments.

Experiment		Feature Exclusion and Dimension Reduction				Survival Outcome			Supplementary Experiment	
#	Purpose	CT Slice Thickness	Tumor Size	Principal Component Analysis	Num of Feature Dimensions	OS	CSS	RFS	Correlation to CT Slice Thickness (Chi-square)	Correlation to Tumor Size (C-Statistic)
						(HR (95%CI) and log-rank test)	(HR (95%CI) and log-rank test)	(HR (95%CI) and log-rank test)		
**1**	**Study All Features**	** **	** **	** **	1,160	1.02 (0.59–1.75)	1.04 (0.53–2.01)	1.17 (0.55–2.51)	**<0.001**	0.628
						0.929	0.905	0.674		
**2**	**Study Redundancy Effect**	** **	** **	**×**	89	1.79 (0.98–3.29)	1.95 (0.93–4.08)	2.63 (1.11–6.21)	**<0.001**	0.605
						**0.033**	**0.043**	**0.009**		
**3**	**Study Scanning Parameter Effect**	**×**	** **	**×**	86	2.58 (1.51–4.42)	13.72 (7.12–26.5)	7.98 (3.76–16.9)	0.872	**0.877**
						**0.002**	**<0.001**	**<0.001**		
**4**	**Study Tumor Size Effect**	**×**	**×**	**×**	81	1.74 (1.01–2.99)	2.95 (1.52–5.72)	6.59 (3.09–14.1)	0.188	0.667
						0.0582	**0.007**	1<0.00		

The bold values represent p <0.05 indicates significance. C-index >0.8 indicates high correlation.

In experiment #1, all the features were used to create a radiomics signature without any exclusion. In this situation, the radiomics signature was not associated with any of the survival outcomes (OS, CSS and RFS, p-value >0.05). In experiment #2, the redundant radiomics features were excluded leaving in only the redundancy-controlled radiomics set yielding a radiomics signature that was significantly associated with OS (HR (95% CI) = 1.8 (1.0–3.3), p-value <0.05), CSS (HR (95% CI) = 2.0 (1.0–4.1), p-value <0.05), and RFS (HR (95% CI) = 2.6 (1.1–6.2), p-value <0.05). The radiomics signature in experiment #2 included striking fewer radiomics features (89/1160 = 7.6%). However, a correlation analysis showed that the second radiomics signature had a high correlation with CT scan slice thickness (p-value <0.001) which is an image acquisition parameter that should not be associated with clinical outcomes. This may be attributed to selection bias inherent in retrospective multi-institutional cohort studies when technical parameters are different between institutions, along with inter-institutional differences in tumor stage, grade, or aggressiveness tumors. Thus, in experiment #3, we used a radiomics feature set used in experiment #2 but after further exclusion of CT slice thickness dependent radiomics features. The third radiomics signature continued to be significantly associated with OS, CSS, and RFS (all p-value <0.05) with even higher magnitude of association; HR (95% CI) increasing to 2.6 (1.5–4.4), 13.7 (7.1–26.5), and 8.0 (3.8–17.0), respectively. The high HRs on predicting patients’ outcomes indicated that the third radiomics signature was a powerful prognostic signature, especially on predicting CSS. However, experiment #3 radiomics signature continued to be associated with tumor size which is an information readily available through routine clinical imaging without the need for complex radiomics analysis. In order to render this radiomics signature independent of ccRCC tumor size, in experiment #4 we further excluded radiomics features (from the set used in experiment#3 model) that are highly correlating with tumor size (C-Index = 0.877) to yield a tumor-size independent radiomics signature. Final results showed that the well-controlled radiomics signature from experiment #4 was significantly associated with CSS (HR (95% CI) = 3.0 (1.5–5.7), p-value <0.05) and RFS (HR (95% CI) = 6.6 (3.1–14.1), p-value <0.05) for ccRCC patients, but was not significantly associated with OS (p = 0.06).

**Figure 2:**
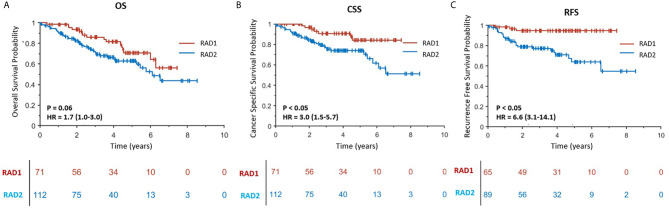
**(A–C)** are Kaplan–Meier curves displaying the association between radiomics phenotypes and patients’ OS, CSS and RFS, respectively. RAD1 and RAD2 represent radiomics phenotypes I and II.

In addition, the associations between confounding factors with survival outcomes were also studied. As shown in **Supplement S1 Figure 1**, there was a significant association between CT slice thickness and patient’s OS (HR (95% CI) = 2.0 (1.2–3.5), p-value <0.01), CSS (HR (95% CI) = 2.0 (1.0–4.0), p-value <0.01) and RFS (HR (95% CI) = 3.6 (1.6–8.0), p-value <0.01), respectively. In **Supplement S1 Figure 2**, there was expected significant association between tumor size and patient’s OS (HR (95% CI) = 2.9 (1.2–5.6), p-value <0.01), CSS (HR (95% CI) = 6.0 (3.0–12.2), p-value <0.01) and RFS (HR (95% CI) = 5.0 (2.3–11.4), p-value <0.01). These two association studies revealed that the patient data in the TCGA-KIRC project were indeed factor-biased data within which real imaging phenotypical signals were suppressed.

### Radiomics Phenotypes

The radiomics feature set used in experiment #4 was our final set to be implemented in constructing final most parasomnias, scanning parameter-independent, and tumor size-independent radiomics signature model classifying the study cohort into two major phenotypes; referred hereafter as radiomics phenotype I (RAD1) and radiomics phenotype II (RAD2). Demographics, pathological characteristics, clinical parameters, and gene mutation status are presented in **Table 4**. There was no statistically significant difference between RAD1 and RAD2 clusters, except in regard the AJCC staging; almost three quarters of patients with RAD1 radiomic signature had stage I tumor while less than half of patients in RAD2 cluster had stage I disease (72% vs 40%, p-value <0.01). No significant difference between the two radiomics phenotypes in terms of gene mutation status as can be seen in **Table 4**.

**Table 4:** Demographic, clinical, pathological, and genetic characteristics of the final radiomics phenotypes.

Patient characteristics		Radiomics Phenotype I (Low-risk, n = 71)	Radiomics Phenotype II (High-risk, n = 112)	p
**Age, year**		**62 (± 12)**	**59 (± 11)**	**0.148**
**Gender**				**0.886**
** **	**Female**	**24 (34%)**	**38 (34%)**	
** **	**Male**	**47 (66%)**	**74 (66%)**	
**Race**				**0.330**
** **	**White**	**68 (96%)**	**108 (96%)**	
** **	**Others**	**3 (4%)**	**4**	
**Tumor grade**				**0.227**
** **	**G1**	**1 (1%)**	**0 (0%)**	
** **	**G2**	**31 (44%)**	**41 (37%)**	
** **	**G3**	**31 (44%)**	**48 (43%)**	
** **	**G4**	**8 (11%)**	**23 (21%)**	
**AJCC TNM staging**				<0.01**
** **	**Stage I**	**51 (72%)**	**45 (40%)**	
** **	**Stage II**	**1 (1%)**	**13 (12%)**	
** **	**Stage III**	**13 (18%)**	**35 (31%)**	
** **	**Stage IV**	**6 (8%)**	**19 (17%)**	
**Distant Metastasis**				**0.267**
** **	**M0**	**65 (92%)**	**95 (85%)**	
** **	**M1**	**6 (8%)**	**17 (15%)**	
**VHL mutation**				0.068*
** **	**Positive**	**43 (68%)**	**57 (53%)**	
** **	**Negative**	**20 (32%)**	**51 (47%)**	
** **	**Not available**	**8 (−)**	**4 (−)**	
**PBRM1 mutation**				**0.207**
** **	**Positive**	**15 (24%)**	**37 (34%)**	
** **	**Negative**	**48 (76%)**	**71 (66%)**	
** **	**Not available**	**8 (−)**	**4 (−)**	
**SETD2 mutation**				**0.843**
** **	**Positive**	**6 (10%)**	**8 (7%)**	
** **	**Negative**	**57 (90%)**	**100 (93%)**	
** **	**Not available**	**8 (−)**	**4 (−)**	
**BAP1 mutation**				**0.746**
** **	**Positive**	**8 (13%)**	**8**	
** **	**Negative**	**55 (87%)**	**100**	
** **	**Not available**	**8 (−)**	**4 (−)**	
**KDM5C mutation**				
** **	**Positive**	**6 (10%)**	**2 (2%)**	0.055*
** **	**Negative**	**57 (90%)**	**106 (98%)**	
** **	**Not available**	**8 (−)**	**4 (−)**	

Values are presented as n (%) for categorical variables and mean (± std) for continuous variables. **indicates high significance with p<0.05, and *indicates weak significance with a p-value

between 0.05 and 0.10.

RAD1 radiomics phenotype included 71 patients and RAD2 phenotype included 112 patients. RAD1 cluster was reflective of the less aggressive ccRCC, in comparison to RAD2 cluster, consistently associated with lower AJCC cancer stage and with better cancer-specific and recurrence-free survival as reflected in **Figure 2**. In terms of overall survival, RAD1 tended to have better survival also but the association was not statistically significant. The most striking divergence of survival is noticed in the recurrence-free survival; Cox-regression hazard ratio of RAD2 vs. RAD1 was HR (95% CI) = 6.6 (3.1–14.1), p-value <0.05.

## Discussion

In this study, we demonstrated a proof of concept to remove redundant, CT slice thickness-dependent (biased), and tumor-size dependent radiomics features towards building a concise radiomics signature in patients with ccRCC. Furthermore, we demonstrated that the final most parsimonious radiomics signature model stratified this multi-institutional cohort into two major radiomics phenotypes that are significantly different by AJCC staging, CSS, and RFS. However, the radiomics signature model was not associated with genetic mutation status nor with any other available patient or tumor characteristic. In this study we have demonstrated how radiomics models can be negatively impacted by confounders (like CT slice thickness) and tumor size (a previously proven prognosticator) leading to false/overvalued signatures. As shown in **Figure 1**, the addition of the controlling procedure after the feature extraction is a supplement to the existing standard radiomics guideline (9), and should be helpful for medical image analysis whose data were usually relatively small and with high heterogeneity of imaging protocol.

Our analysis framework followed an ablation study paradigm, *i.e.*, investigating factors sequentially, from feature redundancy to imaging parameters (CT slice thickness) to tumor size in order to evaluate the effect of each factor to the final radiomics signature. For the feature redundancy, we have shown that there was a large redundancy existing within the radiomics feature set. A raw feature set containing 1,160 features could be efficiently represented by only 89 dimensions of principal components, which represent 7.6% of the original radiomics features. The reason for the existence of redundancy is because feature extractors, including other widely used extractors (20) (e.g., PyRadiomics (38) and IBEX (37)), were based on a number of basic feature extraction algorithms (*e.g.*, Wavelet features (42), Gray-Level Co-occurrence Matrix (GLCM) features (43), etc.), which contained multiple tunable parameters aiming to extract the features in multi-scales for the sake of not missing any valuable image patterns (33). Thus, it is highly recommended that the removal of feature redundancy be the first step when initializing a radiomics analysis. It’s also notable that unsupervised machine-learning methods, such as non-parametric principal component decomposition (39) and unsupervised hierarchical clustering (10), were recommended for redundancy removal and radiomics signature building. Compared to the supervised methods, the unsupervised machine-learning methods generally have a lower risk on overfitting the problem, because little or no prior knowledge is needed for the learning parameters.

For image acquisition parameters, our study showed that ccRCC patients imaged with different slice thicknesses were associated with significantly different survival outcomes, which is not biologically plausible and certainly is attributed to inherent bias in retrospective studies. The patients with thicker CT scan slices thickness were of more aggressive tumors when compared to patients with thinner CT scan slices (See **Supplement S1 Figure 1(A)**, thick vs. thin slice thickness group was of HR of recurrence (95% CI) = 3.6 (1.6–8.0), p <0.01). We believe this apparent association is because institutions that contributed ccRCC to the TCGA and TCIA with thicker CT slices happened to be contributing ccRCC tumors with more aggressive behavior (i.e. larger tumors with higher stage of disease). If the effect of slice thickness on radiomics features is not attended to and controlled for, we would have committed an error by producing a radiomics signature that is dependent on the slice thickness of the CT scan and therefore completely false.

ccRCC tumor size has long been identified as an important prognosticator and it is easily measured on routine abdominal imaging without the need for advanced processing or radiomics. Our study demonstrated that tumor size-dependent radiomics features may exaggerate the clinical utility of radiomics and may mask the real/tumor size-independent radiomics clinical utility. Size independent radiomics features are reflective of tumor textural heterogeneity will ultimately provide additional prognostic information separate from tumor size measurement which is routinely implemented clinically (*e.g.*, clinical staging for kidney cancer (44), RECIST 1.1 (45)). In this study, we introduced a method to remove the effect of tumor size from the radiomics signature models built to yield a size-independent radiomics signature with more valuable input into the tumor internal environment.

In summary, there were two main findings in our work: in retrospective multi-institutional imaging data with heterogenous techniques, image acquisition parameters could lead to false radiomics signatures while size-dependent radiomics may yield overvalued clinical utility of radiomics signature. Unfortunately, there is still suboptimal awareness of these two pitfalls in radiomics literature, although some researchers have tried to establish quality assurance criteria for radiomics study (46).

For image acquisition parameters, most of previous studies on the effect of image acquisition parameters focused on studying feature reproducibility and model generalization. Such studies could only result in conclusions that the heterogeneity in image acquisition parameters decreased the reproducibility and performance of radiomics signatures. Our study, for the first time, showed that the effect of image acquisition parameters could be severe enough to lead to false biologically unplausible association. Attention to and control for imaging acquisition parameters that are influential of radiomics is of crucial importance in retrospective studies, especially multi-institutional ones.

For tumor size, the risk of overvalued radiomics signature induced by tumor-size-effect was mainly caused by a large portion of radiomics features that were basically ‘mixture’ features, which characterized tumor size and intratumor imaging pattern simultaneously, such as Gray-Level Run Length Matrix (GLRLM) (47) and Gray-Level Size Zone Matrix (GLSZM) (48). The contribution weights between tumor size and image patterns to the final feature value were variable depending on the specific tumor phenotypes. Unfortunately, in most of radiomics feature extraction packages, such as PyRadiomics, IBEX, etc., the effect of size on ‘mixture’ features was not well studied (33). Thus, the size effect in ‘mixture’ feature may be easily overlooked and may lead to an over-valued size-dependent radiomics signature. The prognostic information from such a signature will overlap with the prognostic information already retrieved by measuring tumor size. For example, Mattea et al. (15) tested a radiomics signature previously shown to have predictive values on survival outcome among head and neck cancer patients, but eventually, this radiomic signature was found to be a surrogate for tumor size.

The limitations of our work include the following points. First, the number of patients in the TCGA-KIRC project was relatively small and there were no further external data to validate our final radiomics signature. Second, except for slice thickness, other image acquisition parameters, such as scanning mode and reconstruction kernel, were not studied because most of the CT scanning (85%, see **Table 1**) were performed on scanners manufactured by the same vender with similar smooth reconstruction kernels. Fortunately, since the TCIA is a rapidly developing open research community supported by National Cancer Institute, it is promising that more and more projects such as the TCGA-KIRC will be created/improved and available in near future. Third, using PCD for dimension reduction could lead to losing of features’ original physical quantification and make the modeling a black box which is difficult for interpret. Finally, in this study, we only used single-modal imaging and unsupervised machine learning algorithms for modeling, however, as the data increase in future, we could investigate multimodal imaging and supervised machine learning algorithms which have shown promising results in recent years (49–53).

## Conclusion

In this paper, we demonstrated that a radiomics signature could be negatively impacted by multiple factors, including radiomics redundancy from large-scale feature extraction, biases from image acquisition parameters, and underlying dependency to established clinical prognosticator (tumor size). Proper attention to and control for these pitfalls are needed to guarantee a reliable, reproducible, and clinically relevant radiomics signature. Our work used the prediction of survival outcomes in ccRCC patients as an example. In our study, the final most concise, slice thickness independent, and tumor size-independent radiomics signature stratified multi-institutional retrospective cohort of ccRCC into two distinct phenotypes that are significantly different in tumor stage, CSS and RFS.

## Data Availability Statement

The data we used were downloaded from the TCGA-KIRC project, which is publicly available in the TCIA dataset (https://wiki.cancerimagingarchive.net/display/Public/TCGA-KIRC).

## Ethics Statement

The studies involving human participants were reviewed and approved by dataset downloaded from The Cancer Genome Atlas Program supported by National Cancer Institute. The patients/participants provided their written informed consent to participate in this study.

## Author Contributions

LLu: Conceptualization, methodology, formal analysis, and writing (original draft). FA: Conceptualization, methodology, data curation, and writing (extensive review and editing). OA: Resource, data curation and funding acquisition. LLuk: Data curation. XG: Software. HY: Data preparation. JY: Writing (review and editing). AH: Data curation. LS: Conceptualization, discussion, and funding acquisition. BZ: Conceptualization, discussion, and funding acquisition. All authors contributed to the article and approved the submitted version.

## Conflict of Interest

The authors declare that the research was conducted in the absence of any commercial or financial relationships that could be construed as a potential conflict of interest.
