# Supplementary material for: Uncontrolled Confounders May Lead to False or Overvalued Radiomics Signature: A Proof of Concept Using Survival Analysis in a Multicenter Cohort of Kidney Cancer
Source: Front Oncol. 2021 May 27;11:638185. doi: 10.3389/fonc.2021.638185 (PMC8191735; doi:10.3389/fonc.2021.638185)

***Supplement S1. Survival outcome analysis based on CT slice thickness and tumor size***


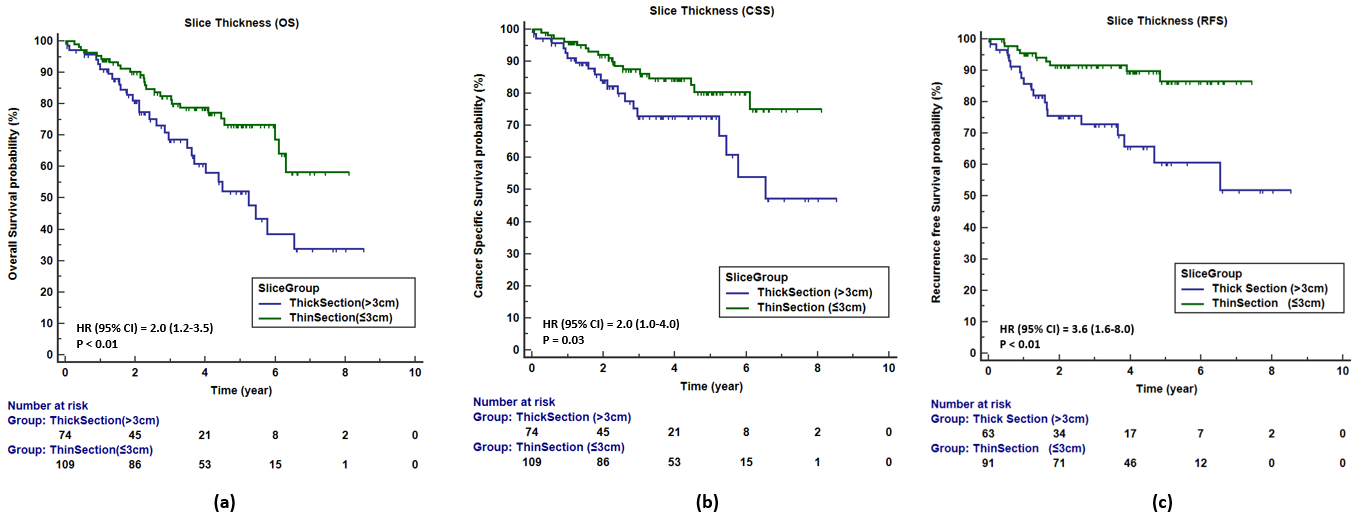


**Supplementary Figure 1. (a) (b) and (c) are Kaplan–Meier curves displaying the association between slice thickness and patients’ OS, CSS and RFS, respectively.**


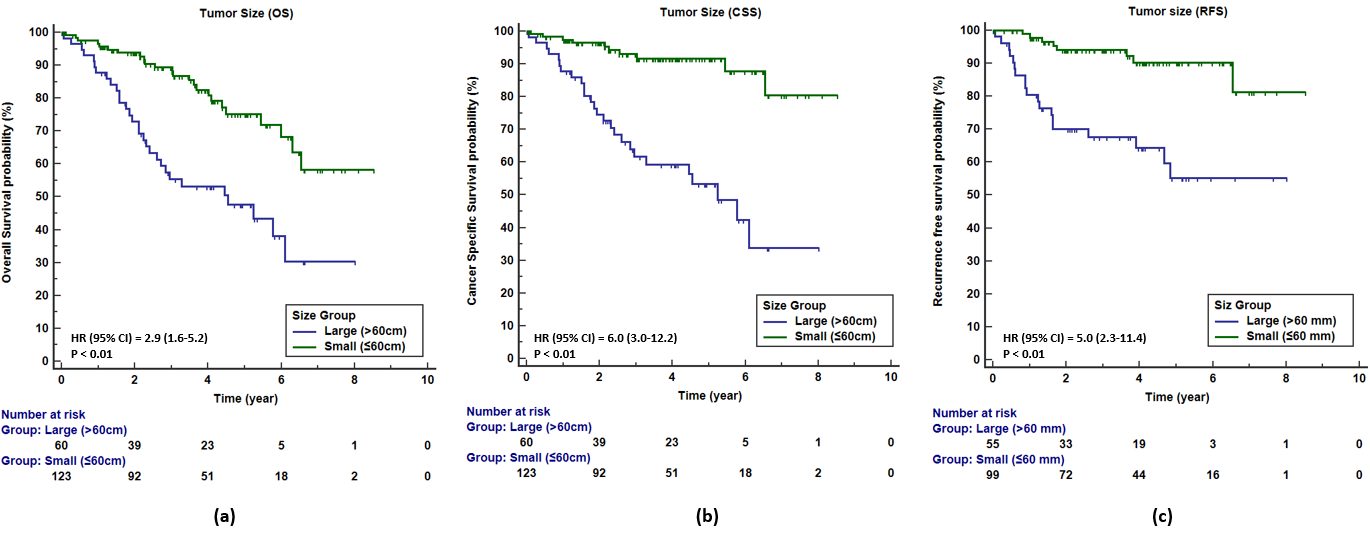


**Supplementary Figure 2. (a) (b) and (c) are Kaplan–Meier curves displaying the association between tumor size and patients’ OS, CSS and RFS, respectively.**

***Supplement S2. CT scan characteristics based on data source cites.***


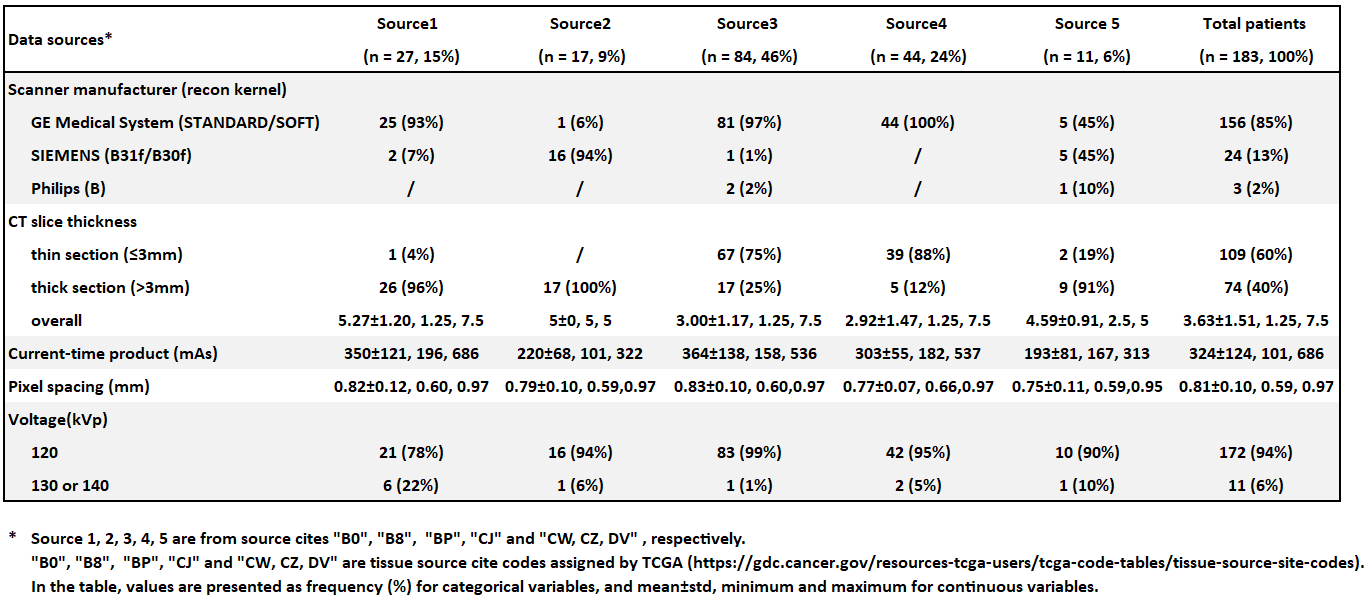

Supplement: Supplementary file 1 [file DataSheet_1.docx]
